# Endobronchial Valves for Recurrent Hemoptysis Secondary to Aspergilloma: A Case Report and Literature Review

**DOI:** 10.7759/cureus.79685

**Published:** 2025-02-26

**Authors:** Parnia Khamooshi, Karthik Vijayan, Michael DiRico, Hiren Mehta

**Affiliations:** 1 Pulmonary and Critical Care Medicine, University of Florida, Gainesville, USA

**Keywords:** aspergilloma, chronic pulmonary aspergillosis, endobronchial valves, hemoptysis, interstitial lung disease

## Abstract

Aspergilloma is a common cause of hemoptysis and can result in significant morbidity and mortality in patients with structural lung disease. While surgical resection is the gold standard for treating aspergilloma, many patients are not suitable candidates for surgery. In this report, we explore the use of endobronchial valves (EBVs) as a non-surgical alternative for managing recurrent hemoptysis in a patient with interstitial lung disease (ILD) and a left upper lobe aspergilloma. Despite previous treatments, including antifungals and radiation, the patient experienced persistent hemoptysis and was not a candidate for either bronchial artery embolization or lobectomy. An EBV was implanted in the affected lung segment, resulting in the resolution of the hemoptysis without valve-related complications on the six-month follow-up. While the use of EBVs for hemoptysis management has been reported in a few cases, this is, to our knowledge, the first report in a patient with ILD. This approach presents a viable non-surgical alternative in complex cases where conventional treatments have proven ineffective or are not feasible.

## Introduction

Aspergilloma, a type of mycetoma, is a form of chronic pulmonary aspergillosis (CPA) characterized by developing a fungal nodule or mass inside pre-existing pulmonary cavities. Tuberculosis, non-tuberculosis mycobacteria, and allergic bronchopulmonary aspergillosis are the most common risk factors for CPAs. However, CPAs also occur in the setting of other cystic or cavitary lung diseases such as emphysema, prior pneumothorax, treated lung cancer, and rarely interstitial lung disease (ILD) [1,2].

Most patients with aspergilloma are asymptomatic. However, a minority of patients can have varying amounts of hemoptysis, making aspergilloma an important cause of massive and recurrent hemoptysis [3]. Hemoptysis in aspergilloma results from the erosion of blood vessels within pulmonary cavities due to the fungal ball, with bleeding originating from bronchial and chest wall arteries supplying the cavity [3]. Among patients with CPA, patients with ILD as the underlying disease were reported to have significantly poor prognosis [2]. Here, we describe a case of recurrent hemoptysis in a patient with ILD treated non-surgically with an endobronchial valve (EBV).

## Case presentation

A 60-year-old African American woman with a history of bronchiectasis and ILD from 10 years prior presented to our institution for the management of recurrent hemoptysis for the last two years. She also reported shortness of breath on heavy exertion, which had remained stable in the last 10 years. She did not have a history of tobacco smoking or any other recreational drugs and had no known environmental toxin exposure.

**Figure 1:**
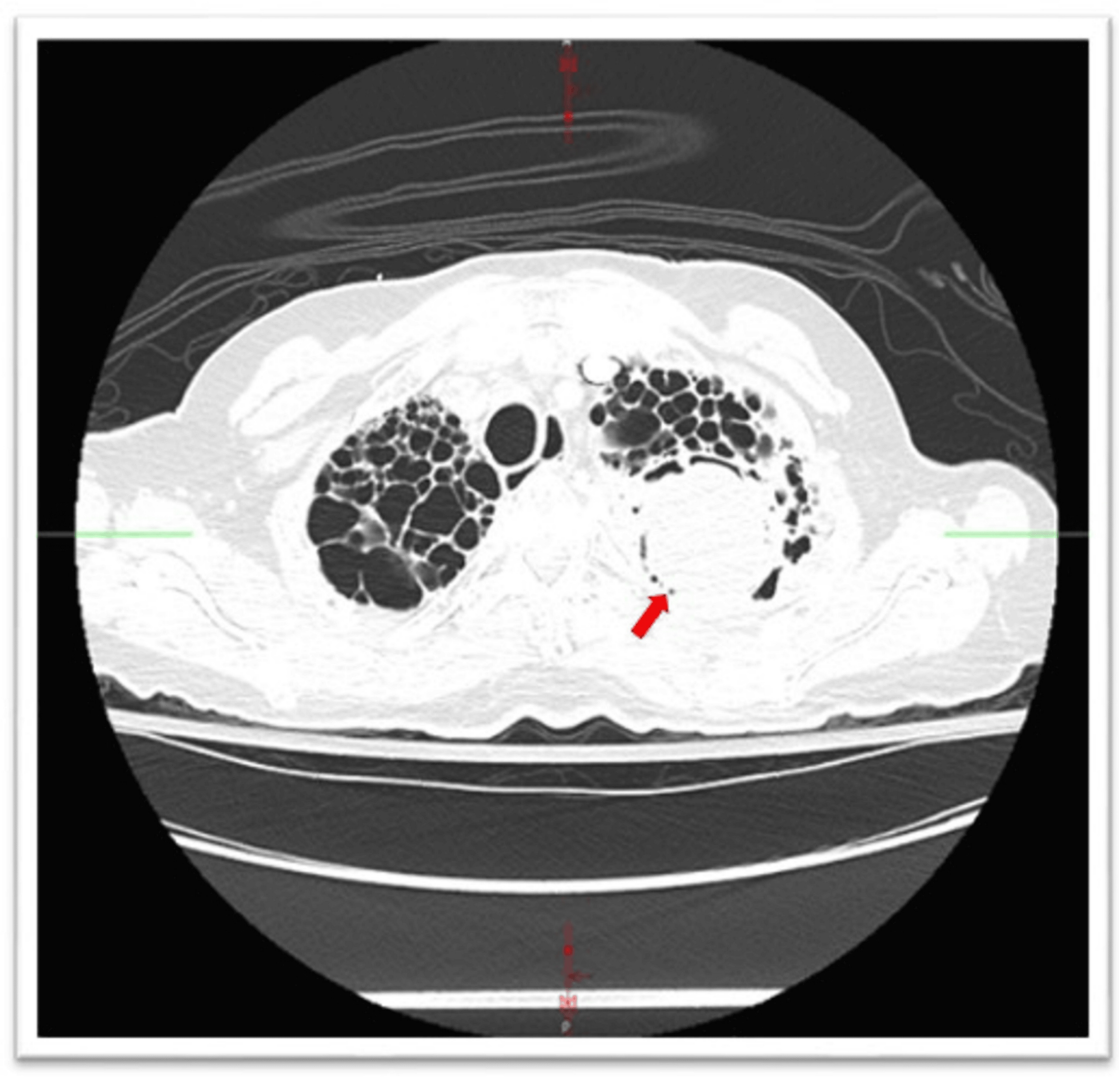
Axial section of the chest CT with contrast showing extensive cystic lesions at bilateral upper lobes and a left upper lobe aspergilloma inside the cavitary lesion (arrow).

Physical examination was significant for thin body habitus with a body mass index of 18 kg/m^2^ and decreased breath sounds in bilateral upper lobes. Computed tomography (CT) of the chest was significant for bilateral upper lobe cystic lesions, left upper lobe (LUL) mass inside a cavitary lesion, and bilateral hilar calcification (Figure 1).

She had undergone extensive workup at different academic institutions in the United States for evaluation of her ILD, including negative autoimmune and hypersensitivity pneumonitis panels, which were unrevealing for specific etiology. A surgical wedge resection of her right upper lobe (RUL) showed a foreign body giant cell reaction containing polarizable foreign substance and was negative for organisms or malignancy. Per multiple ILD multidisciplinary discussions, she was given a diagnosis of unknown foreign body exposure and/or sarcoidosis and was treated with varied doses of prednisone ranging between 5 and 30 mg daily for the last 10 years. She was treated with varying doses of prednisone (ranging from 5 to 30 mg daily) over the past 10 years, during which time her dyspnea remained stable, and imaging showed stable upper lobe cysts.

She had over 18 hospital admissions for hemoptysis in the past two years and had undergone multiple bronchoscopic examinations, which revealed varying degrees of bleeding in the LUL, ranging from blood-tinged secretions to active bleeding, with no evidence of endobronchial lesions. Infectious workup results, including positive cultures and polymerase chain reaction for *Aspergillus fumigatus* from bronchoalveolar lavage and positive serum IgG for *Aspergillus*, confirmed the diagnosis of aspergilloma as the underlying cause of her hemoptysis.

**Figure 2:**
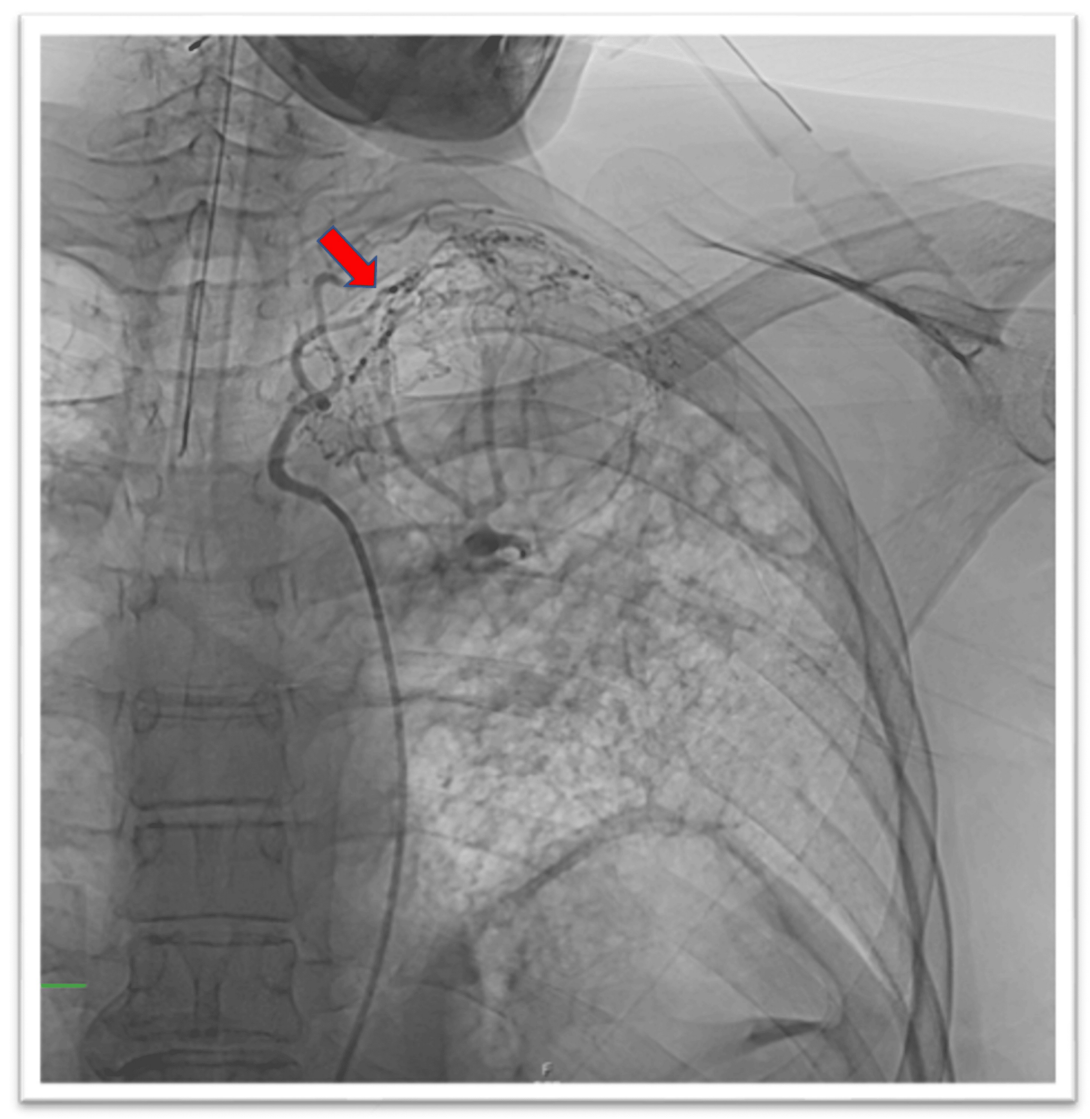
Bronchial artery angiogram showing arterial supply of aspergilloma (arrow).

The patient’s hemoptysis persisted despite multiple treatment modalities, including long-term itraconazole 200 mg given twice daily, two rounds of radiation therapy, and continuation of systemic steroids. A bronchial artery angiogram was performed (Figure 2) showing the arterial supply of the aspergilloma.

However, embolization was not an option due to the bronchial artery anatomy, putting the patient at an increased risk of blockage of the superior spinal artery with the potential to cause anterior spinal ischemia. Furthermore, the patient was not a surgical candidate for resection due to the extent of the disease and limited pulmonary reserve, as evidenced by pulmonary function tests showing a restrictive pattern with decreased diffusion capacity (forced vital capacity (FVC): 53% predicted, forced expiratory volume in one second (FEV1): 57% predicted, FEV1/FVC: 85%, total lung capacity: 51% predicted, and diffusing capacity of the lung for carbon monoxide: 32% predicted).

**Figure 3:**
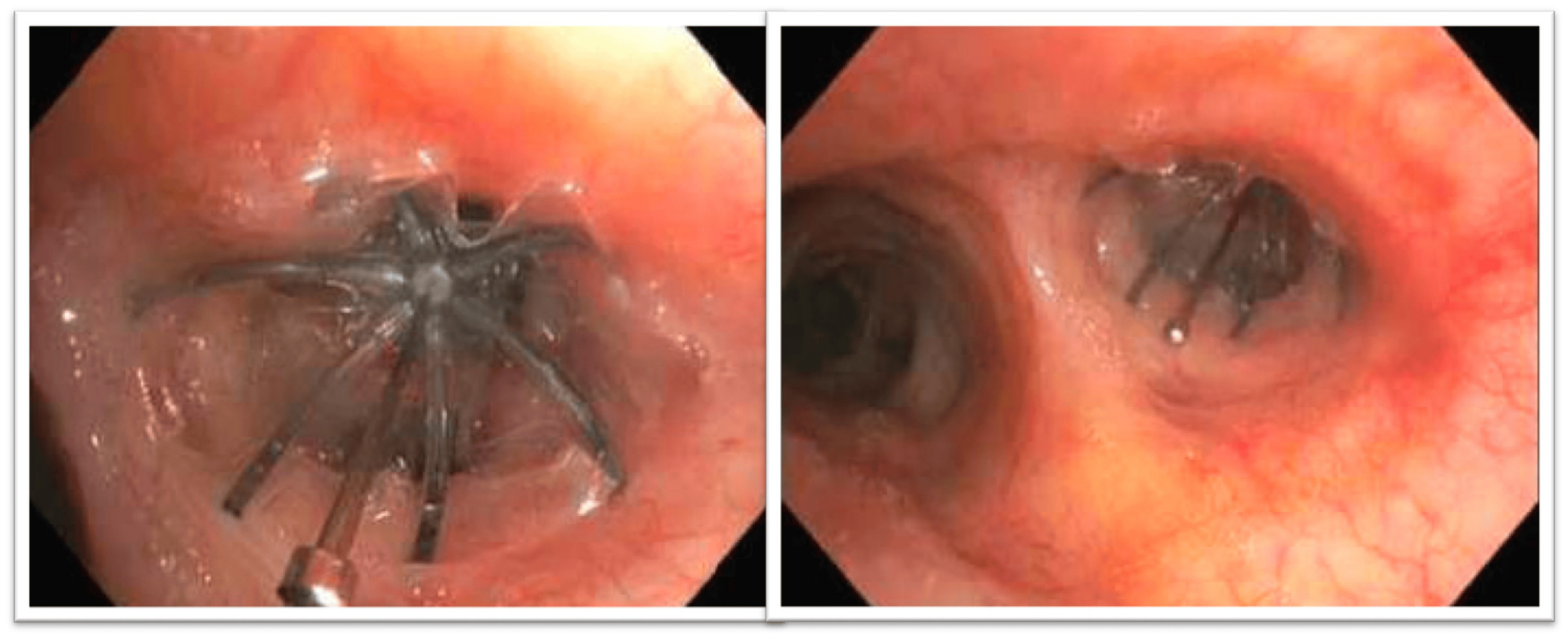
Left upper lobe with the Spiration valve in place (left). Left mainstem bronchus with left upper lobe Spiration valve in place (right).

She was transferred to our institution following a prolonged hospitalization without success in temporizing her massive hemoptysis. A diagnostic fiberoptic bronchoscopy was performed, during which a small amount of blood-tinged secretions were noted within the LUL bronchus. To manage her ongoing hemoptysis, we placed a 9 mm Spiration® valve (SVS, Olympus, Redmond, WA, USA) in the proximal LUL, covering the superior division and lingular bronchi (Figure 3).

The patient remained hemoptysis-free on the six-month follow-up and had no complications related to the valve.

## Discussion

Surgical resection is the most definitive treatment in patients with hemoptysis due to focal aspergilloma and, in fact, may decrease overall mortality [4]. However, there may be a significant risk of morbidity and mortality associated with the procedure, and many patients have contraindications to surgery due to poor respiratory reserve, multiple aspergillomas, and other comorbidities [4]. Because of this risk or contraindications, less invasive measures are often sought first.

If an episode of hemoptysis can be managed with supportive care, antifungal medications have been utilized to treat aspergillomas and prevent further exacerbations. Numerous antifungals have been attempted for this purpose and have been administered systemically, inhaled, or applied topically to the lesion. While they may delay the recurrence of hemoptysis, they are usually not curative, even when administered over a prolonged course of one year [4,5]. Tranexamic acid has been utilized inhaled, orally, or intravenously, with variable results [4,6].

Bronchial artery embolization (BAE) is a highly effective alternative treatment or temporizing measure for a focal source of hemoptysis. However, approximately half of patients who undergo BAE will have recurrent hemoptysis [4,6]. One feared complication of BAE is spinal cord ischemia, leading to permanent paraplegia or paraparesis. This complication occurs due to inadvertent embolization of spinal arteries branching from bronchial or intercostobronchial arteries [7]. This anatomical variation is a contraindication of BAE, as was identified in our patient.

While not frequently utilized, stereotactic body radiotherapy is also an option for focal disease [6,8]. Radiotherapy causes perivascular necrosis and occludes circulation. This method is commonly used to manage hemoptysis associated with malignancy [9]. However, data on the utility of this method in hemoptysis due to aspergilloma is limited [6,8].

A few case reports have reported the use of EBVs for hemoptysis management [10-14]. EBVs were primarily developed for bronchoscopic lung volume reduction for emphysema and the management of bronchopleural fistulas. There are two commercially available EBVs in the United States, namely, Zephyr® EBV (Pulmonx, Redwood City, CA, USA) and Spiration® Valve System. In theory, EBVs could be helpful in managing hemoptysis by causing local atelectasis, which leads to tamponade, clot formation, and decreased blood flow at the bleeding site [12-14]. However, this utility of EBVs has not yet been widespread and remains off-label.

Successful use of EBVs for managing hemoptysis has been reported in a few benign and malignant pathologies [10-14]. Among benign cases, most were associated with aspergilloma in the setting of tuberculosis. Solis et al., Koegelenberg et al., and Lalla et al. described using EBVs in patients with recurrent hemoptysis that were not amenable to surgical resection or with unsuccessful BAE attempts [10-12]. Frey et al. reported using EBVs in a critically ill patient with septic emboli leading to persistent hemoptysis. In this case, BAE could not be performed due to poor visualization of the bronchial artery during the procedure [13]. Spiration and Zephyr EBVs were successfully used in these cases, with hemoptysis-free outcomes lasting up to six months post-procedure. In malignancy-associated hemoptysis, Patel et al. described the successful management of two cases with EBVs. The first involved RUL lung squamous cell carcinoma, and the second involved esophageal cancer with mediastinal invasion, resulting in hemoptysis from the RUL. Both cases were effectively managed using Spiration EBVs placed in the RUL [14].

The Spiration EBV was selected for our patient based on its umbrella-shaped design and absence of a fish mouth, which was thought to promote clot formation more effectively than the Zephyr valve. Nonetheless, the literature remains lacking in consensus regarding the optimal valve choice.

Common complications associated with EBV placement include pneumothorax, respiratory failure, pneumonia, valve migration, and hemoptysis [15]. In our case, the patient did not experience any complications related to the EBV, and similar cases have reported no adverse effects.

One notable advantage of utilizing EBVs for hemoptysis management is their ease of removal, should complications arise or after successful hemorrhage control, especially if the underlying cause is expected to be reversible. The literature presents varied approaches to EBV removal; some studies opted not to attempt valve removal [14], while others performed removal after six months [10,11]. Notably, the earliest reported EBV removal occurred two weeks after placement in a single case [13]. In our case, we also chose not to remove the valve, as the underlying cause of the patient’s hemoptysis is unlikely to resolve in the near future. However, given the limited available evidence, establishing the optimal timing for valve removal to avoid complications associated with EBVs warrants further investigation.

## Conclusions

This report highlights EBV as a viable, minimally invasive alternative for managing hemoptysis in patients who are poor surgical candidates or have failed conventional treatments. It offers effective symptom control and may serve as a bridge to definitive management. However, further research is needed to evaluate its long-term safety and efficacy in a broader patient population. A multidisciplinary approach should be considered before using EBVs for this indication to ensure optimal patient selection and treatment outcomes.

## References

[1] Smith NL, Denning DW (2011). Underlying conditions in chronic pulmonary aspergillosis including simple aspergilloma. Eur Respir J.

[2] Yamakawa H, Nishizawa T, Ohta H (2022). Patient background and prognosis of chronic pulmonary aspergillosis in fibrosing interstitial lung disease. Medicine (Baltimore).

[3] Corr P (2006). Management of severe hemoptysis from pulmonary aspergilloma using endovascular embolization. Cardiovasc Intervent Radiol.

[4] Tashiro M, Takazono T, Izumikawa K (2024). Chronic pulmonary aspergillosis: comprehensive insights into epidemiology, treatment, and unresolved challenges. Ther Adv Infect Dis.

[5] Sharma S, Kumar R, Ish P, Mahendran AJ, Gupta NK, Gupta N, Madan M (2023). Clinical utility of intrabronchial antifungal instillation in a complicated case of chronic pulmonary aspergillosis: case report and systematic review of literature. Infez Med.

[6] Lang M, Lang AL, Chauhan N, Gill A (2020). Non-surgical treatment options for pulmonary aspergilloma. Respir Med.

[7] Panda A, Bhalla AS, Goyal A (2017). Bronchial artery embolization in hemoptysis: a systematic review. Diagn Interv Radiol.

[8] Koch A, Schanne DH, Günther G, Aebersold DM, Elicin O (2023). Stereotactic body radiotherapy for recurrent hemoptysis due to chronic pulmonary aspergillosis: a case report and systematic review of the literature. Strahlenther Onkol.

[9] Gaito S, Hughes C, Woolf D, Radhakrishna G (2019). Radiotherapy in the control of bleeding from primary and secondary lung tumours. Br J Hosp Med (Lond).

[10] Koegelenberg CF, Bruwer JW, Bolliger CT (2014). Endobronchial valves in the management of recurrent haemoptysis. Respiration.

[11] Lalla U, Allwood BW, Sinha Roy S, Irusen EM, Koegelenberg CF (2017). Endobronchial valve used as salvage therapy in a mechanically ventilated patient with intractable life-threatening haemoptysis. Respiration.

[12] Solis Solis AJ, Centeno Clemente CA, Rosell Gratacos A (2024). Endobronchial valves in the management of recurrent hemoptysis: a case report. Open Respir Arch.

[13] Frey JW, Postigo M, Pitts LR (2023). Endobronchial valve placement as salvage therapy in the management of hemoptysis. J Bronchology Interv Pulmonol.

[14] Patel B, Abi-Fadel D, Rosenheck J, Bartter T, Boujaoude Z, Abouzgheib W (2019). Endobronchial valves for treatment of hemoptysis. J Bronchology Interv Pulmonol.

[15] Low SW, Swanson KL, Lee JZ, Tan MC, Cartin-Ceba R, Sakata KK, Maldonado F (2022). Complications of endobronchial valve placement for bronchoscopic lung volume reduction: insights from the Food and Drug Administration Manufacturer and User Facility Device Experience (MAUDE). J Bronchology Interv Pulmonol.

